# Origins of truncated supplementary capsid proteins in rAAV8 vectors produced with the baculovirus system

**DOI:** 10.1371/journal.pone.0207414

**Published:** 2018-11-15

**Authors:** Lionel Galibert, Adrien Savy, Yohann Dickx, Delphine Bonnin, Bérangère Bertin, Isidore Mushimiyimana, Monique M. van Oers, Otto-Wilhelm Merten

**Affiliations:** 1 Genethon, Evry, France; 2 FinVector Oy, Kuopio, Finland; 3 Synpromics Ltd., Edinburgh, United Kingdom; 4 University of Eastern Finland, A.I. Virtanen Institute for Molecular Sciences, Kuopio, Finland; 5 Wageningen University and Research, Laboratory of Virology, Wageningen, the Netherlands; Wuhan Bioengineering Institute, CHINA

## Abstract

The ability to produce large quantities of recombinant Adeno-Associated Virus (rAAV) vectors is an important factor for the development of gene therapy-based medicine. The baculovirus/insect cell expression system is one of the major systems for large scale rAAV production. So far, most technological developments concerned the optimization of the AAV *rep* and *cap* genes in order to be expressed correctly in a heterologous system. However, the effect of the baculovirus infection on the production of rAAV has not been examined in detail. In this study we show that the baculoviral cathepsin (v-CATH) protease is active on several (but not all) rAAV serotypes, leading to a partial degradation of VP1/VP2 proteins. Subsequently, we identified the principal v-CATH cleavage site in the rAAV8 capsid proteins and demonstrated that the cleavage is highly specific. The proteolytic degradation of VP1/VP2 AAV capsid proteins reduces the infectivity of rAAV vectors but can be prevented by the use of a baculovirus vector with a deletion of the *chiA*/*v-cath* locus or by addition of the E64 protease inhibitor during production. Moreover, the codon optimization of AAV *cap* performed for several serotypes and originally aimed at the removal of potential alternative initiation codons, resulted in incorporation of additional forms of truncated VP1 into the rAAV capsids.

## Introduction

Gene delivery vectors derived from Adeno-Associated Virus (AAV) are widely used for development of treatments against a range of rare genetic diseases. In 2002, the first rAAV vector based on the baculovirus-insect cell expression system was made available [1]. Three recombinant baculoviruses were used, one encoding the rAAV-genome, a second carrying the AAV capsid (*cap*) genes and a third one encoding the AAV large and short replicase (*rep*) genes. The second generation rAAV production system combined *rep* and *cap* in a single recombinant baculovirus [2]. This latter system showed higher levels of the AAV VP1 protein in the rAAV particles and an improved genetic stability of the baculovirus vectors, in particular, for the baculovirus carrying the *rep* and *cap* genes (reviewed in [3]). Today, one of the main challenges for the further development of baculovirus-based rAAV-technology lies in the optimization of this expression system with respect to quantity (titer) and quality of rAAV vectors manufactured at a large scale.

One of the problems when producing certain rAAV serotypes in insect cells, as exemplified here for rAAV8, is that, in addition to the expected capsid proteins VP1 (81.6 kDa), VP2 (66.6 kDa), and VP3 (59.9 kDa), supplementary VP protein bands are observed in Western blot aimed against AAV capsid proteins on purified rAAV particles [4]. Up to now it is not clear whether these additional polypeptides are degradation products of the VP proteins mentioned above or whether they result from transcription or translation initiation at alternative start sites in the *cap* sequence. In the current study, we analyzed the effect of the chitinase (ChiA) and cathepsin (v-CATH) proteins encoded by the baculovirus vector on the integrity of the rAAV capsid proteins. Chitinase and cathepsin are working in tandem during baculovirus infection of lepidopteran larvae to achieve dissemination of the occluded form of the baculovirus progeny [5,6]. The viral chitinase is a glycohydrolase that mediates the correct folding of v-CATH [7]. The baculovirus v-CATH shares features with proteins in the cathepsin B [8] and cathepsin L [5] families. Baculovirus-induced cell lysis releases chitinase and the active form of v-CATH [9], resulting in degradation of the chitin-exoskeleton and liquefaction of the internal organs of the insect [6]. In the genome of the baculovirus commonly used for recombinant protein expression and rAAV production, *Autographa californica* multiple-capsid nucleopolyhedrovirus (AcMNPV), c*hiA* and *v-cath* are flanking genes [10]. The removal of the *chiA*/*v-cath* locus from the AcMNPV genome has previously been shown to improve the integrity of the secreted version of the *Theileria parva* sporozoite surface protein P67 [11] and to enhance the productivity of, for instance, HSP90, Polo Like Kinase 1, and the phosphatase-and-tensin-homolog protein [12].

We hypothesized that the baculovirus v-CATH protease may also degrade one or more rAAV capsid proteins when synthesized in insect cells, leading to diminished capsid integrity and a reduced potency as viral vector. In order to test this hypothesis, we produced rAAV8 vectors using either the standard AcMNPV bacmid system [13] or a bacmid system with a deletion of the *chiA/v-cath* locus. We compared the rAAV capsid protein profiles and evaluated their ability to transduce mammalian cells. We confirmed involvement of v-CATH in rAAV8 capsid degradation using the E64 protease inhibitor. In addition, we examined whether the codon optimization of the *cap* sequences performed originally in the laboratory of R. Kotin [2] similarly to the optimisation of the *rep* sequence and designed to remove potential translation initiation sites from the VP1 unique domain, was responsible for the generation of additional truncated VP1 proteins that were also incorporated into rAAV capsids.

## Material and methods

### Baculovirus gene deletions

Deletion of *v-cath* and *chiA* from the AcMNPV bacmid was performed in the *E*. *coli* DH10Bac strain containing the AcMNPV bacmid [13] that was transformed with the pKD46 plasmid [14] to allow Lambda Red recombination. The *v-cath* and *chiA* DNA region to be eliminated from the AcMNPV genome was chosen according to Kaba et al. [11]. The PCR product necessary for the *v-cath* and *chiA* gene inactivation, encoding the chloramphenicol acetyl transferase (*cat*) gene flanked by lox66/lox72 sequences [15] was generated with primers CC-KO-F and CC-KO-R (S1 Table) using the pcrTOPO-lox-CAT-lox plasmid as template [16]. Homologous recombination to inactivate the genes was performed according to Marek et al. [16] and assessed using the primers chitinase-105625F and cathepsin-107849R (S1 Table). The *cat* gene marker was removed from the *v-cath/chiA* null bacmid (AcbacΔCCΔcat) as described before [16] and the resulting bacmid AcbacΔCC was verified through PCR and sequencing, using the previously mentioned primers. Inactivation of *p10* coding sequence in AcbacΔCC was performed in the same manner, with PCR products generated with primer pair p10-KO-F/p10-KO-R (S1 Table). The *cat* resistance marker was again removed. Verification of correct gene inactivation was performed using PCR and sequencing with primer pair p10-118725-F/p10-119259-R (S1 Table). The latter gene inactivation led to the v-*cath/chiA/p10* null bacmid (AcbacΔCCΔp10).

### Insertion of AAV *rep/cap* genes and recombinant AAV genome into bacmid by transposition

The rAAV sequence encoding the γSGC transgene was previously described [17] and sub-cloned in the pFBDual plasmid (Invitrogen) between SnaBI and StuI sites using NEB restriction enzymes. The pFBD-mSeAP plasmid encoding the *murine secreted alkaline phosphatase* reporter gene (mSeAP) controlled by the CMV promoter and flanked by the Flop/Flop Inverted Terminal Repeats (ITRs) of AAV2 was generated likewise in pFBDual using the same restriction sites. The pFBD-Rep2-Cap8 construct has been received from Rob Kotin and its sequence was verified. In this construct, the *cap8* gene was replaced by enzymatic restriction with *cap1 or cap6* sequences originating from wild type AAV, serotypes 1 and 6, respectively. The *cap*2, *cap rh10*, and wild type *cap8* (GenBank: AF513852.1) sequences were synthetized (Genewiz), *cap rh10* was originally designed by Achille François (UMR1089, Nantes, France), and *cap9* by Rachid Benchaouir (Université de Versailles Saint-Quentin-en-Yvelines, France). The *cap* gene sequences are listed in S1 Fig. The resulting pFBDual vectors were sequenced and used to transform *E*. *coli* DH10Bac cells containing either the wild type bacmid (WT), AcbacΔCC or AcbacΔCCΔp10 and the helper plasmid pMON7124 [13]. Efficient recombination in the bacmid genomes was assessed by PCR using primer pairs M13pucF/R and M13pucF/Genta (S1 Table).

### Cell line, baculovirus and rAAV production

Sf9 cells (Gibco) were grown in suspension culture at 27°C in 500 mL of SFM900III medium (Invitrogen) in 1L Corning Erlenmeyer Flasks (Ref: 431147) under rotation speed of 170 rpm on INFORS HT Celltron shaker. Baculoviruses were generated from recombinant bacmids according to the guidelines of the Bac-to-Bac protocol and were amplified in 150 mL of suspension cultures of Sf9 cells at a density of 10^6^ cells per mL in 250 mL Corning Erlenmeyer Flasks (Ref: 431144). rAAV productions were performed by dual infection of baculoviruses harboring the recombinant AAV genome (γSGC or mSeAP) and the AAV *rep/cap* genes, each at a MOI of 0.05 (PFU titer) in 150 mL of Sf9 cell culture seeded at 10^6^cells.mL^-1^ in 250 mL Erlenmeyer Flasks. At 96 h p.i. 1 mL of the total culture was recovered for direct quantification of rAAV production prior to purification. Concerning rAAV productions performed using the WT baculovirus, E64 protease inhibitor (Sigma #E3132) was added to a subset of cell cultures to a final concentration of 50 μM at different times post-infection (0h, 24h, 48h, 72h, 96h, and post cell lysis for rAAV recovery). wtAAV2 has been produced as described by Zeltner et al. [18], with the pDG2 plasmid [19] and the WT AAV2 sequence (NC_001401.2) obtained by gene synthesis from the Genewiz company and cloned into the pGRG25 plasmid [20].

### AAV2 VP1 silencing, deletion of the N-terminal part of VP1

VP1 silencing in pFBD-rep2-cap2 was performed by site-directed mutagenesis with Pfu Turbo Cx Hotstart DNA Polymerase (Agilent) (Start ACG modified to ACT) using primer pair VP1-KO-F/R (S1 Table). A 411 bp fragment of the 5’ end of the VP1 ORF including its start codon was deleted by digesting the pFBD-rep2-cap2 plasmid using BssHII and EcoNI restriction enzymes.

### rAAV purification and characterization

100 mL of bulk culture of rAAV of serotypes 1, 2, 6, 8, 9, rh10, were treated for 2.5 hours at 27°C with 0.5% Triton-X-100. Treated bulk cultures were then filtered through a Pall Preflow (DFA3001UBC) for rAAV of serotypes 1, 6, 8 and rh10; and through a Sartorius Sartobran 300 0.45–0.2 μm (5231307H5) for rAAV2; or through a Merck Millipore Millipak 100 Durapore 0.22 μm (MPGL1GCA3) for rAAV9. All rAAV cultures except serotype 9 were purified using 50 mL of AVB sepharose medium (GE Healthcare) according to [2]. rAAV9 particles were purified on 1 mL of POROS CaptureSelect AAV9 affinity matrix, with elution from the matrix performed according to [2]. Purified rAAV vectors were dialyzed against PBS (Gibco) using Millipore Ultracel 100 KDa units (UFC910024).

### Determination of the rAAV genome titer

A quantitative PCR assay was performed directly on the total culture samples or purified rAAV samples to determine the rAAV titer (viral genome copies per mL of culture). Viral DNA was extracted directly from the bulk or from purified samples using the MagNA Pure DNA and viral NA small volume kit (MagNA Pure 96, Roche), respectively. qPCR titrations were performed against a reference on a Roche LightCycler 480II using primers and probe ITR-F/R/P (S1 Table) for rAAV genome titration.

### SDS-PAGE and Western blot analysis

Purified rAAV vectors were run on SDS-PAGE Bis-Tris 4–12% gels (Nu-PAGE, Invitrogen), and either directly Coomassie stained or transferred to iBlot gel transfer stack nitrocellulose (Invitrogen) prior to immuno detection. Mouse IgG1 (derived from clone B1 recognizing a C-terminal region; Progen) was used in a 1/250 dilution as antibody against AAV VP of the studied serotypes. The Anti Rep mouse IgG1 clone 303.9 (Progen) was used in a 1/100 dilution. Anti VP polyclonal antibody VP51 (Progen) was used in a 1/250 dilution. The secondary antibody was a goat anti-mouse Dye 680 (LI-COR) used in a 1/5000 dilution. Antibody incubations were performed in Odyssey blocking buffer (LI-COR) and colour visualisation was performed on the Odyssey system (LI-COR).

### *In vivo* injection and sample collection

rAAV vectors equivalent to 10^9^ vg in 25 μL of phosphate buffered saline were injected into the left Tibialis Anterior (TA) muscle of 6 weeks old C57bl\6 mice. Four animals were injected for each assayed rAAV vector. Blood samples were collected from the injected mice at 3, 7, 14, 21, 28, 35 days post-injection, for subsequent mSeAP seric quantification. At day 35, the animals were sacrificed and the TA muscles, left and right were collected and frozen before histological, enzymatic assays, and qPCR analysis. All mice were handled according to directive 2010/63/EU on the protection of animals used for scientific purposes. *In vivo* experiments were approved by Genethon´s ethics committee.

### mSeAP detection in serum and in tissues

Samples of 12 μL mouse sera were used to detect mSeAP activity. mSeAP quantification was realised using the Phospha-Light System kit (Applied Biosytems). Samples were read on a Victor II Luminometer apparatus. mSeAP levels were expressed as counts per second (CPS). Statistical analysis has been performed using t-test (Prism, GraphPad).

### Detection of rAAV genome *in vivo*

Total DNA samples were extracted from mice muscles using the FastDNA kit (QBIOgene) on a FastPrep apparatus (QBIOgene). rAAV genome titrations were performed using qPCR as described for purified rAAV above. qPCR on the *titin* gene (gene id: 7273) was performed using primers and probe titin-F/R/P. The *titin* qPCR value was used for normalization of the rAAV genome copies. Statistical analysis, one-way ANOVA followed by Dunnett's Multiple Comparison test and figure drawing were performed using Graphpad.

## Results

### The baculovirus v-cathepsin degrades the VP1 protein of rAAV8 capsids

rAAV8 vectors, encoding the human gamma-sarcoglycan (γSGC) transgene [17], were produced with baculovirus technology using bacmids that harbored deleted or non-deleted *chiA* and *v-cath* genes. Titers of rAAV obtained in the bulk were in the same range for the rAAV produced with the unmodified bacmid system (WT) as for rAAV produced with the Δ-*chiA/v-cath* baculovirus (ΔCC) [2.5 x 10^11^ viral genomes (vg).mL^-1^ ± 1.3 x 10^10^ (std. dev.) versus 1.9 x 10^11^ vg.mL^-1^ ± 3.5 x 10^10^ (std. dev.), as averaged over 3 replicates for both WT and ΔCC].

**Fig 1:**
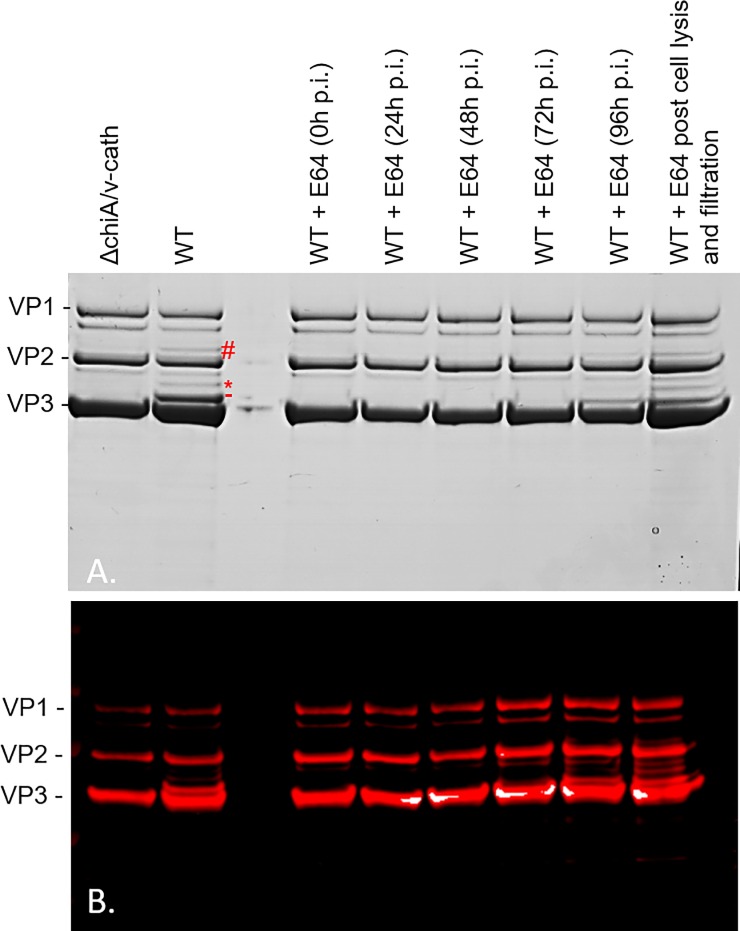
The baculovirus v-CATH protease induces rAAV8 capsid degradation. rAAV8 vectors encoding the γSGC transgene were produced in the baculovirus system with the *v-cath* and *chiA* genes deleted (ΔchiA/v-cath) or with the unmodified bacmid system (WT) and purified using immuno-affinity chromatography. The protease inhibitor E64 was added to the WT production runs at various times post infection (0, 24, 72 or 96h p.i.). The cells were harvested and lysed at 96h p.i. In the final sample E64 was added following harvest, cell lysis and clarification of the cell culture. Samples of 3 x 10^9^ vg of rAAV8 γSGC were loaded on the SDS-PAGE followed by Coomassie staining (A) or by Western blot using Anti VP B1 antibody (B). The red dash (-) pinpoints to the major 61 kDa degradation band originating from VP1/VP2 unique domains. The asterisk (*) indicates a minor degradation band originating from the VP1/VP2 unique domain. The hash (#) indicates minor degradation band originating from VP1 unique domain.

However, when the protein profiles of the purified rAAV8 particles were analyzed for both baculovirus production systems, the products displayed different protein patterns. For both rAAV preparations, the expected capsid proteins VP1 (81.6 kDa), VP2 (66.6 kDa) and VP3 (59.9 kDa) could be observed after SDS-PAGE (Fig 1A). However, in the rAAV8-γSGC produced with the WT baculovirus a major extra polypeptide of 61 kDa was observed just above the VP3 protein (Fig 1A. dash). We also detected two other supplementary polypeptides of lower intensity in the WT preparations. One was found above the major supplementary band of 61 kDa (Fig 1A. asterisk), while the other protein band was observed at a molecular mass larger than the VP2 protein (Fig 1A. hash). The fact that these supplementary bands are only present when the WT baculovirus backbone is used suggests that they are degradation products of the AAV capsid proteins. Western blot analysis of the same samples, using the B1 antibody (Progen), which recognizes the C-terminal part of the three AAV capsid proteins [21] also identified the major extra protein band as well as the two minor supplementary bands observed with the WT baculovirus (Fig 1B). This finding leads to the conclusion that they are N-terminally truncated VP1/VP2 proteins.

To further confirm, that the extra VP proteins identified for rAAV8 produced with the WT baculovirus were the result of degradation of VP1/VP2 proteins by the v-CATH, rAAV8 was produced in the presence or absence of the E64 protease inhibitor. This chemical has been shown before to inhibit the activity of the baculovirus v-CATH [5]. E64 was added at different stages during the baculovirus infection process and the purified rAAV8 vectors were again analyzed by SDS-PAGE. rAAV8 produced in the absence of E64 displayed the extra AAV VP protein bands, while rAAV8 vectors produced in the presence of E64 (at least when E64 was added before 72h p.i.) did not contain these truncated VP proteins (Fig 1A and 1B). The degradation products of the rAAV8 vector accumulated during the time course of rAAV8 production. Maximum degradation was seen upon addition of Triton-X-100 during the cell lysis step prior to purification of the rAAV8 vector at 96 h p.i. (Fig 1A and 1B). With this result, we confirmed that an E64-sensitive protease (i.e. v-CATH) is active at the later stages of the baculovirus infection, especially when cell lysis occurred, and that this protease was involved in the degradation of VP1 and/or VP2 proteins of rAAV8 vectors. Two additional minor polypeptides were present in both the WT and ΔCC preparations that most likely are not the consequence of v-CATH activity, as rAAV8 produced with the Δ-*chiA/v-cath* baculovirus did not prevent their emergence (Fig 1A and 1B). The generation of these proteins could not be precluded by adding E64, indicating they are not generated through v-CATH activity.

### Mechanism of cleavage–non-susceptibility of rAAV8 empty capsids to the v-cathepsin protease

**Fig 2:**
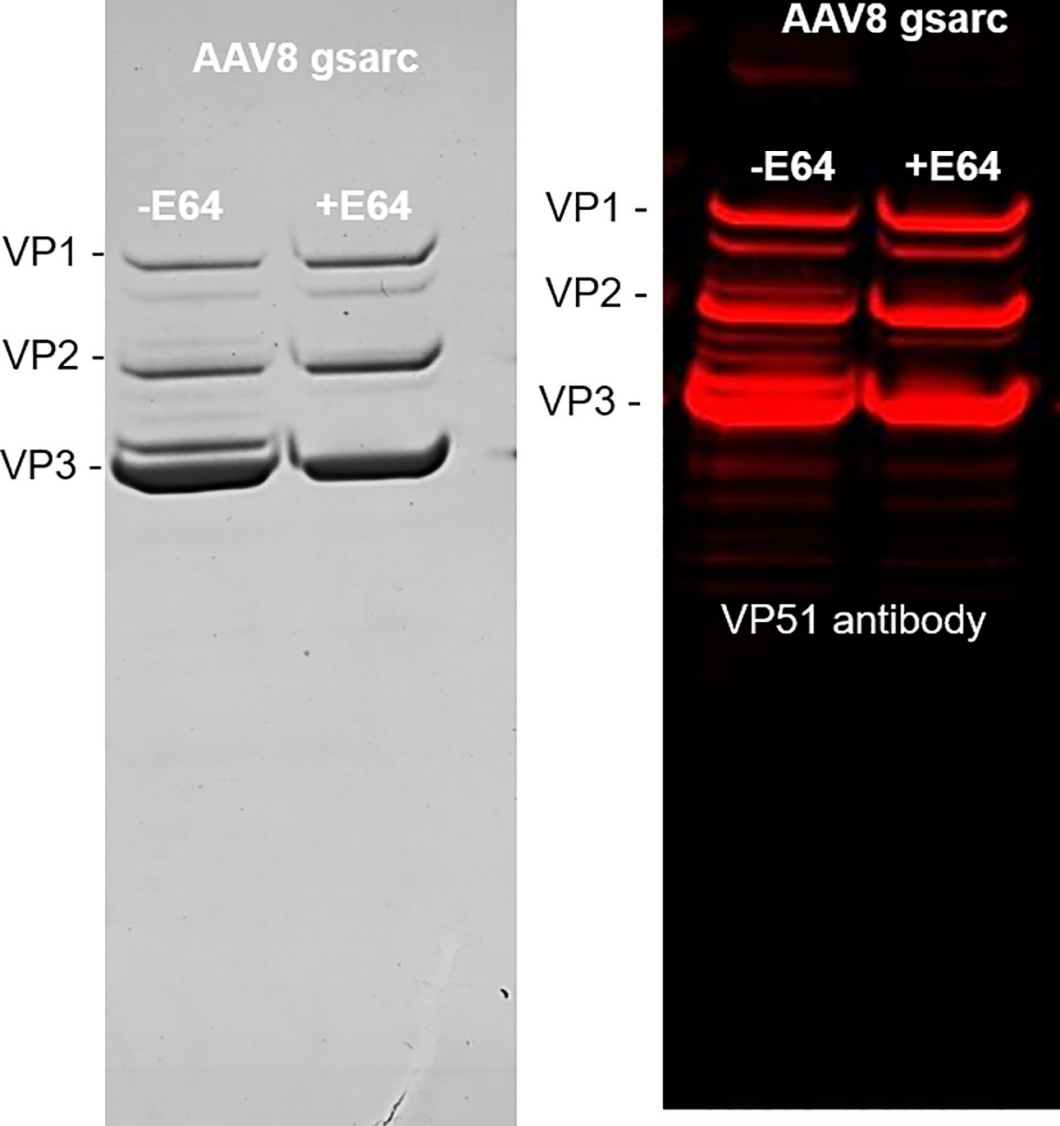
rAAV8 capsid degradation by v-CATH studied with anti-VP polyclonal antibody. rAAV8 vectors encoding the γSGC transgene were produced in an unmodified bacmid system in the presence or the absence of E64 protease inhibitor and purified using immuno-affinity chromatography. Purified vectors were loaded on SDS-PAGE stained with Coomassie blue (left panel). The corresponding Western blot is shown on the right panel.

It was also reported that the N-terminal domain of VP1/VP2 were localized inside empty and full rAAV capsids (at least for serotype 2 [22]) and should not be accessible to the v-CATH. To determine whether the VP1/VP2 N-terminal domains were on the external side of the capsid when cleaved by v-CATH, or inside the capsid but left accessible to the v-CATH, we tried to detect the N-terminal cleavage product of the VP1/VP2 proteins in the purified rAAV particles. For this assay, we used a polyclonal antibody directed against the entire VP1 sequence (Progen VP51, anti-AAV VP1/VP2/VP3), but did not detect any supplementary band at a molecular mass of 21 kDa, which would be expected for the N-terminal v-CATH cleavage product of the VP1 protein (82 kDa), (82 kDa minus the 61 kDa observed above when detecting the C-terminal end). Likewise, we did not detect any supplementary band at 6 kDa, which would complement the C-terminal part of VP2 protein cleaved by v-CATH (67 kDa minus 61 kDa) (Fig 2). We could also not observe any protein with an approximate mass of 21 or 6 kDa size in stained SDS-PAGE gels (Fig 2). The fact that these N-terminal cleavage products are not retained in the rAAV particle points toward the presence of the N-terminus of the VP1/VP2 protein on the external side of the capsid when cleaved by the v-CATH.

**Fig 3:**
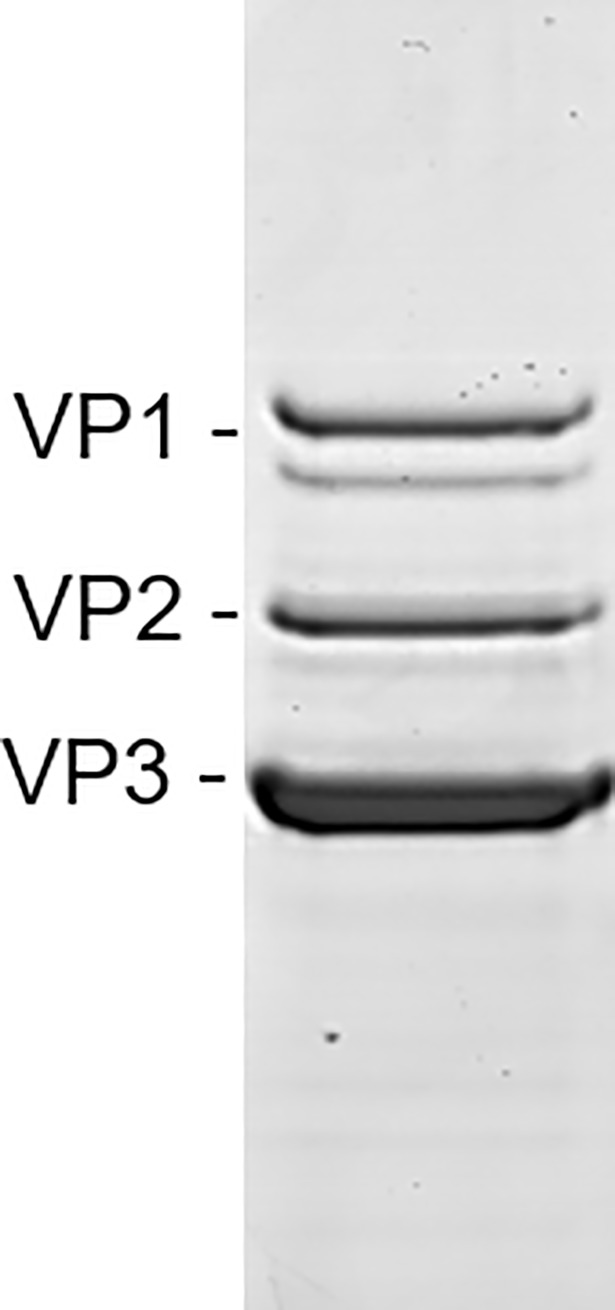
The baculovirus v-CATH protease does not degrade empty rAAV8 capsids. Empty rAAV8 capsids were produced in unmodified bacmid system by infecting Sf9 cells only with Bac-rep/cap baculovirus at MOI 0.05, purified using immuno-affinity chromatography, analyzed on SDS-PAGE and revealed by Coomassie staining.

Furthermore, we were interested to know whether VP1/VP2 would also be (partially) exposed in empty AAV vector particles. Thus, we produced empty rAAV8 capsids by infection of Sf9 cells only with the baculovirus encoding the *rep* and *cap* genes. In contrast to full capsids, the empty rAAV8 capsids did not contain the degradation products (Fig 3) observed when the rAAV8 production was performed in the presence of the baculovirus encoding the rAAV-γSGC genome. Thus, only the rAAV capsids containing recombinant genomes are susceptible to the degradation of the v-CATH protease. This observation strongly indicated that the degradation of the rAAV8 capsid by the v-CATH protease takes place after capsid assembly and rAAV genome packaging, probably in the cells undergoing baculovirus-induced lysis, or in the bulk of the cell culture during harvesting.

### Identification of cleavage sites of the v-cathepsin on rAAV8 capsids

**Fig 4:**
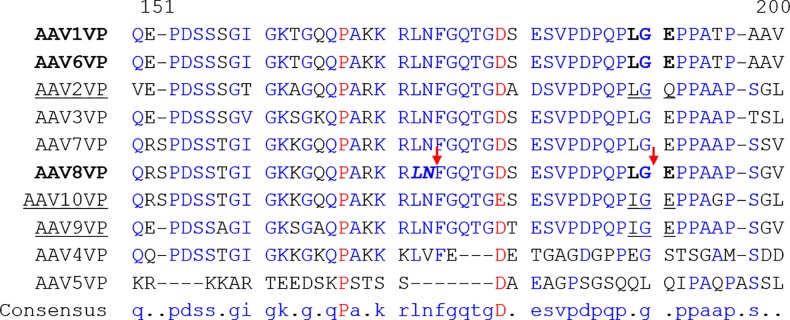
Amino acid sequence alignment of VP proteins of AAV serotype 1 to rh10 at the major cleavage site induced by v-CATH. Amino acid sequence of AAV VP1 proteins of serotype 1 to 9 and rh10 were aligned using multalin software [35]. The display includes amino acids comprised from aa 151 to 200. Amino acids conserved among all studied rAAV serotypes are displayed in red color. Conserved amino acids among several serotypes are displayed in blue color. Gaps introduced into the alignment are represented by dash police. rAAV serotypes (1, 6, 8) cleaved by the baculovirus v-CATH are displayed in bold characters at the major cleavage position in the rAAV VP amino acid sequences. Minor cleavage site happens in rAAV8 following LN amino acids in bold italic police in the figure. Both major and minor cleavage sites by v-CATH in rAAV8 are indicated by vertical red arrows. The amino-acid sequences of rAAV serotypes (2, 9, rh10) not cleaved by v-CATH but corresponding to the amino acid region cleaved for susceptible serotypes are underlined.

To identify the major v-CATH cleavage site, we performed N-terminal sequencing of the 61 kDa degradation product (Fig 1A. dash). The Edman sequencing reaction allowed the identification of the unique amino acid residues Glu_191_-Pro-Pro-Ala-Ala-Pro_196_ at the N-terminal end of this degradation product. The triplets coding for the amino acid motif are located 13 triplets upstream of the AUG (methionine) translation start of VP3. Accordingly, the cleavage in the AAV8 VP1/VP2 proteins happens after the Leu_189_-Gly_190_ residues (Fig 4).

We were also able to identify a second rAAV8 cleavage site that would result in the minor degradation band of 62 kDa (Fig 1A asterisk). In this case the Edman sequencing reaction allowed the identification of the unique amino acid residues Phe_174_-Gly-Gln-Trp-Gly_178_ at the N-terminal extremity, and thus, cleavage had occurred following the Leu_172_-Asn_173_ residues in the VP1/2 unique domain (Fig 4 displays the rAAV8 sequence in this region).

### Several rAAV serotypes are susceptible to the v-CATH protease

**Fig 5:**
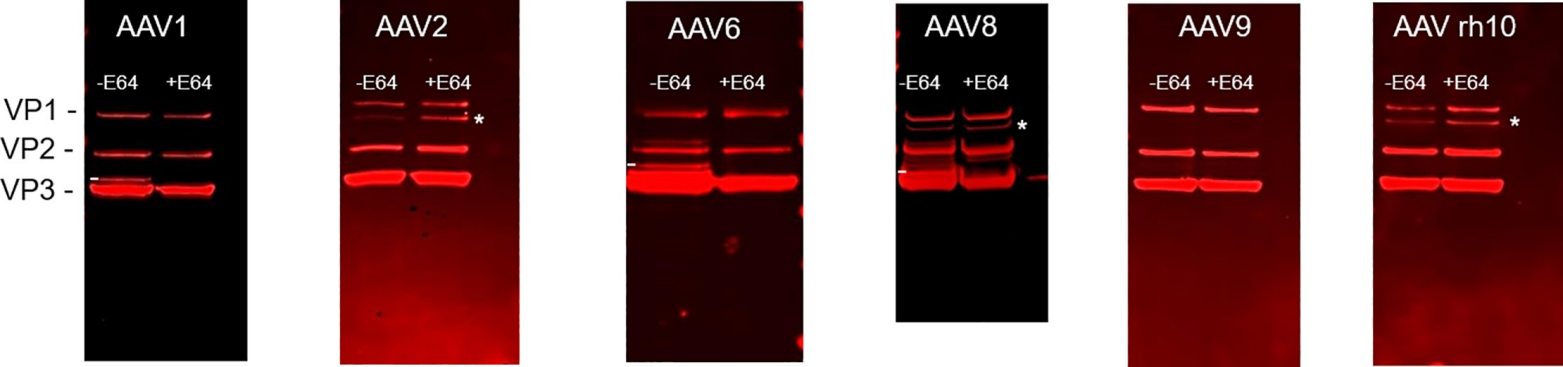
Effect of v-cathepsin protease on various rAAV serotypes. rAAV vectors of serotypes 1, 2, 6, 8, 9, rh10 encoding the γSGC transgene were produced in an unmodified bacmid system in the presence or in the absence of E64 protease inhibitor and purified using immuno-affinity chromatography. VP proteins were revealed by Western blot using Progen B1 antibody. Supplementary bands originating from codon optimization performed in the *cap* genes are marked by an asterisk (*). Supplementary bands originating from v-CATH degradation are marked with a dash (-).

**Fig 6:**
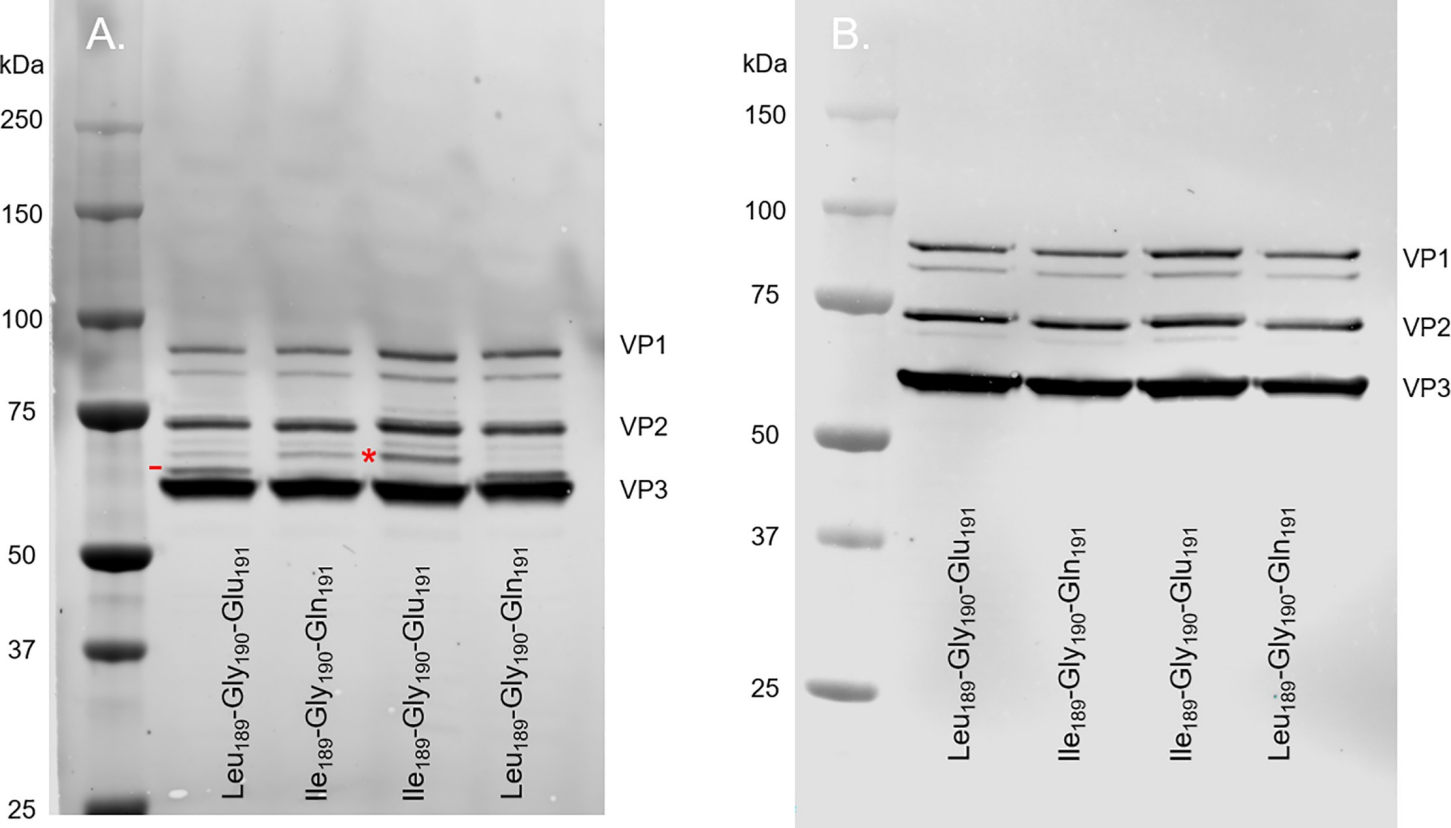
Mutation of V-CATH major cleavage site in AAV8 VP1/2 proteins confirms high specificity of the protease. Based on sequence analysis of several AAV VP serotype sequences, key amino acids Leu_189_ was identified as critical for susceptibility to V-CATH cleavage, while Glu_191_ was suspected to be involved in v-CATH susceptibility. Mutated rAAV8 (on *cap8* codon optimized sequence) encoding the γSGC transgene were produced in an unmodified bacmid system, purified and studied by Western blot using anti-VP B1 antibody. From left to right, rAAV8 samples with amino acid sequence Leu_189_-Gly_190_-Glu_191_ (as found in WT *cap8* sequence); Ile_189_-Gly_190_-Gln_191_ (double mutant); Ile_189_-Gly_190_-Glu_191_ (single mutant); Leu_189_-Gly_190_-Gln_191_ (single mutant). Major degradation product of the VP1/2 unique domain by V-CATH is signaled by a dash. Secondary degradation product of VP1/2 unique domain by V-CATH is signaled by *. AAV8 was produced in absence (A) or presence (B) of E64 protease inhibitor, purified and then analyzed.

Knowing that the amino acid sequence Leu_189_-Gly_190_-Glu_191_ surrounds the major cleavage site in rAAV8 and that this motive is conserved at the same position in VP1/VP2 of several other AAV serotypes, we investigated whether the v-CATH could cleave other rAAV serotypes in a comparable way. We produced rAAV encoding the γSGC transgene using the unmodified (WT) baculovirus backbone to express the capsid genes of serotypes 1, 2, 6, 9, and rh10. The productions were performed with or without addition of the E64 protease inhibitor. Serotypes 1 and 6 share the amino acid sequence Leu-Gly-Glu in the VP1 and 2 proteins with AAV8 (Fig 4). Western blot analysis directed against the VP proteins was performed with the purified capsids of serotype 1 and 6 (Fig 5, marked by a white dash). In the absence of E64, we could visualize the supplementary protein band of 61 kDa on Western blots just above the VP3 protein and similar to what was previously observed for the VP profile of AAV8. Equally, this supplementary band disappeared when the E64 protease inhibitor was added during the rAAV production. Thus, VP1 proteins of AAV capsids of serotypes 1 and 6 were also degraded by the baculovirus v-CATH. For the rAAV of serotypes 2, 9 and rh10 no degraded VP protein was visible on Western blots, irrespective of the presence or absence of E64 during production (Fig 5). This result indicates that the rAAV capsids of serotypes 2, 9 and rh10 are not susceptible to the baculovirus protease. For the VP of rAAV2, instead of Leu-Gly-Glu, the amino acid sequence at the corresponding site is Leu-Gly-Gln, and for the serotypes 9 and rh10 the amino acid sequence is Ile-Gly-Glu (Fig 4). So, the serotypes in which these motives differ slightly (in that a leucine is replaced by an isoleucine residue, or the glutamic acid is replaced by glutamine), are not cleaved by the v-CATH protease showing a high level of selectivity for cleavage site recognition. To further confirm the role played by Leu_189_ and Glu_191_ in the susceptibility to v-CATH protease we mutated those amino acids in the AAV8 capsids and produced rAAV8 using the WT baculovirus. Mutation of Leu_189_ to Ile leading to Ile_189_-Gly_190_-Glu_191_ resulted in abolished proteolytic activity at this position in VP1/2, evidenced by absence of the supplementary protein band above VP3 (Fig 6A. Lane 3.) (The major cleavage site by v-CATH is marked by a dash on Fig 6A. Lane 1). Although this mutation reduced or abolished this cleavage by v-CATH, a more intense proteolytic activity seemed to arise at the alternative v-CATH sensitive site present more towards the N-terminus in the VP1/2 proteins (Fig 6A. Lane 3, marked by an asterisk). The double mutation of both Leu_189_ to Ile and Glu_191_ to Gln, resulting in Ile_189_-Gly_190_-Gln_191_, displayed a similar pattern as the single mutation Ile_189_-Gly_190_-Glu_191_. On the other hand, mutation of Glu_191_ to Gln leading to Leu_189_-Gly_190_-Gln_191_ did not change the susceptibility to v-CATH protease (Fig 6A. Lane 4). Thus, this single mutation of Leu_189_ to Ile_189_ is able to abolish degradation of rAAV8-γSGC capsids at this particular site. The v-CATH origin of the supplementary bands was again confirmed by the absence of degradation when E64 was added during rAAV8 production (Fig 6B).

### Origin of the other supplementary bands in the rAAV8 capsid

During the experiments to determine the susceptibility of different rAAV serotypes to the v-CATH protease, we observed an additional protein band on the Western blots of the rAAV serotypes 2, 8 and rh10, with an estimated size of 77 kDa, a little smaller than the VP1 protein (Fig 5, marked by an asterisk). Interestingly this supplementary polypeptide was also identified as a N-terminally truncated VP protein as it was recognized by the anti-VP B1 antibody specific for the C terminus (Fig 5, marked by an asterisk). This truncated protein was found irrespective of the presence or absence of the E64 protease inhibitor. Hence, the appearance of this polypeptide was not a result of degradation by v-CATH proteolytic activity. This additional band was not observed for the rAAV of serotypes 1, 6 and 9. The origin of this supplementary peptide can be traced back to the codon optimizations by introducing 46 silent mutations in the first 300 nucleotides (nt) of the *vp1* sequence [2]. The objective of these codon optimizations was to remove all potential initiation codons in the *cap8* sequence that could have allowed initiation of translation in-between the VP1 and VP2 start codons. In the *cap2* and *cap rh10* coding sequences 11 and 31 nt were changed in the first 300 nt, respectively, for similar purposes. Finally, the *cap9* coding sequence was also codon optimized, but on a much shorter span. In fact, 4 point mutations were introduced in the first 76 nt following the initiation codon of the VP1 protein. We observed that the truncated VP1 protein was only seen with the serotypes for which the *cap* sequence was codon optimized between nt 76 and 300 (Fig 5, marked by an asterisk). Interestingly, in the work published by Cecchini et al. [4] the additional band could also be seen, when a codon optimized sequence of *cap6* was used. Since we used the natural, non-optimized *cap6* coding sequence (except for the modification of the ATG initiation codon to ACGallowing a certain level of ribosomal scanning to initiate translation of VP2, the Assembly Activating Protein [23] and VP3 [2]) no supplementary truncated VP1 band was seen (Fig 5). This was also the case for serotype 1. As the codon optimizations introduced silent mutations in respect to the encoded protein, we assume that the additional VP band found in serotypes 2, 8 and 10 is the result of alternative translation initiation and not a degradation of VP1.

**Fig 7:**
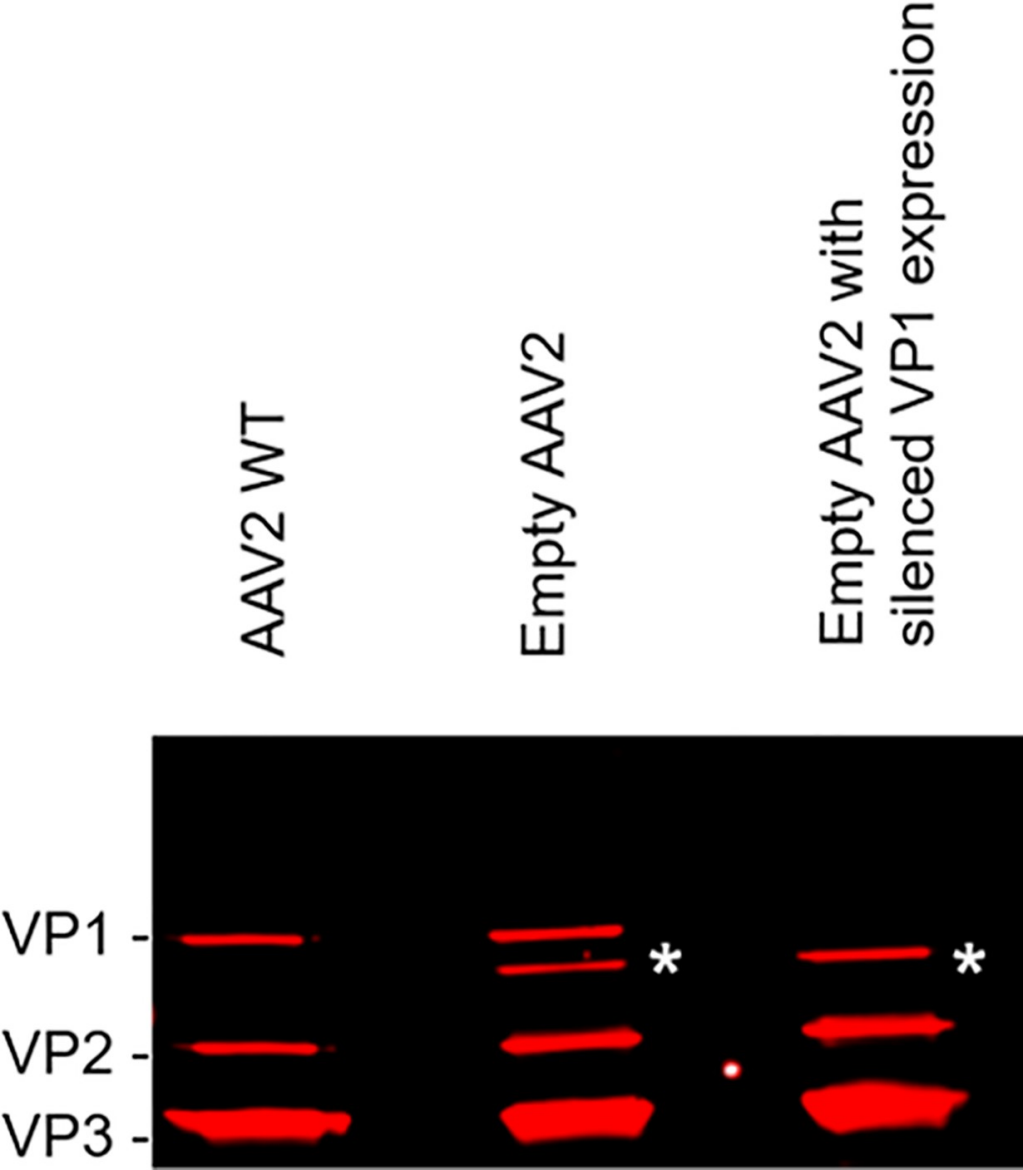
Supplementary truncated VP1 band is a product of translation and not a degradation product. Origin of supplementary truncated VP1 protein band (signaled on the figure by *) was studied by Western blot using anti-VP B1 antibody. (1) **AAV2 WT**. 5 x 10^9^ vg of AAV2 WT produced by transfection of HEK 293 cells. (2) **Empty AAV2**. AAV2 empty capsids produced with baculovirus deleted of *chiA*/*v-cath* genes, with the *cap2* gene codon optimized. (3) **Empty AAV2 with silenced VP1 expression**. AAV2 empty capsids produced with baculovirus deleted of *chiA*/*v-cath* genes, with *cap* gene codon optimized, including silencing of VP1 alternative start codon (ACG → ACT).

**Fig 8:**
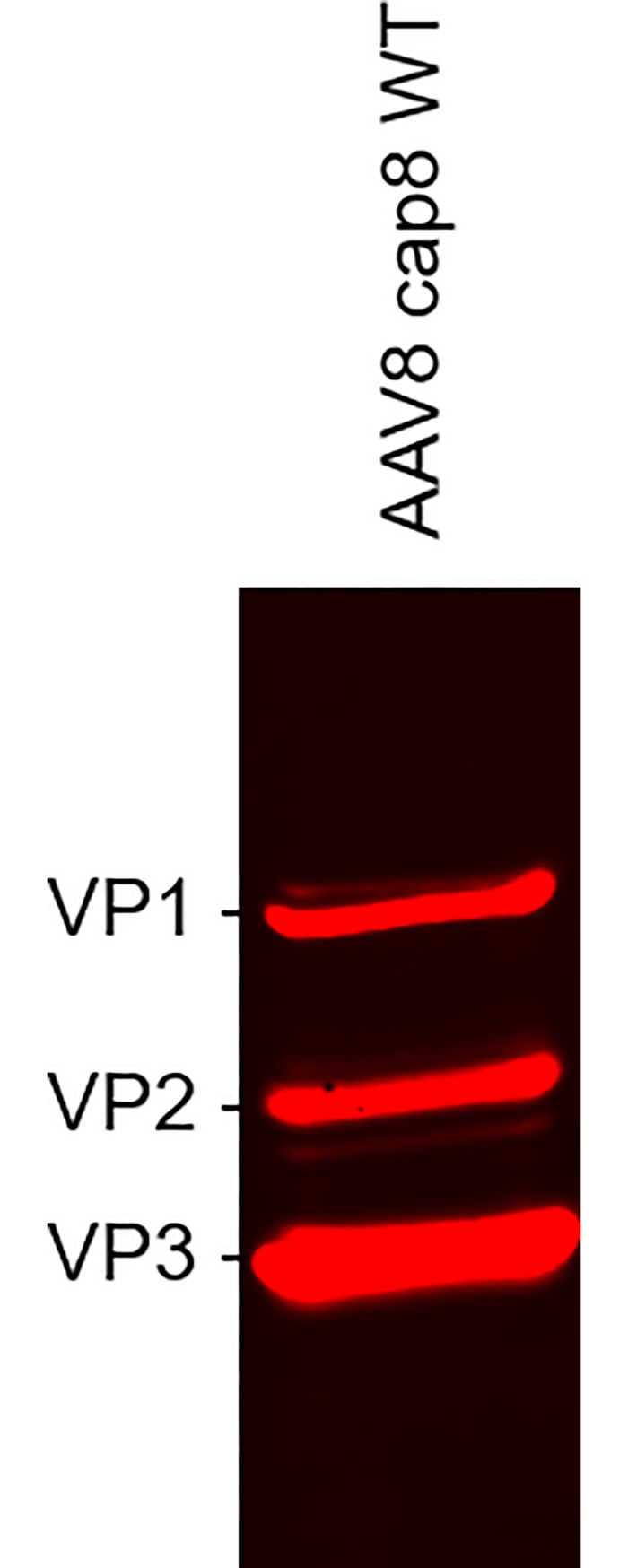
WT *cap8* gene does not lead to additional truncated VP product in rAAV8 capsid. rAAV8 was produced in a bacmid system with the *v-cath* and *chiA* genes deleted. WT *cap8* gene sequence was used without any of the codon optimization. Western blot against VP proteins was used on bulk samples of Sf9 cells infected with baculovirus deleted of *chiA*/*v-cath* genes encoding WT *cap8* gene.

We then produced and purified rAAV2 capsids using the codon optimized *cap*2 ORF described above to further support this conclusion. In one version, we silenced the ACG initiation codon of VP1 to ACT. We successfully suppressed the translation of VP1 in this construct, but we were still able to visualize the supplementary truncated VP1 protein (Fig 7). We thus confirmed that this supplementary band is not a degradation product of VP1 but originated from translation initiation downstream of the VP1 ACG codon in the *cap2* sequence. To further confirm that codon optimizations in the VP1 sequence led to the production of a supplementary truncated form of VP1, we produced rAAV8 vectors with the WT sequence of VP1 also conserving the non-optimal VP1 start codon (ACG). In this configuration, the rAAV8 vectors produced did not display the additional truncated VP1 protein band (Fig 8).

### Degradation of VP1 protein by the v-CATH in AAV8 capsids reduces the rAAV infectivity *in vivo*

**Fig 9:**
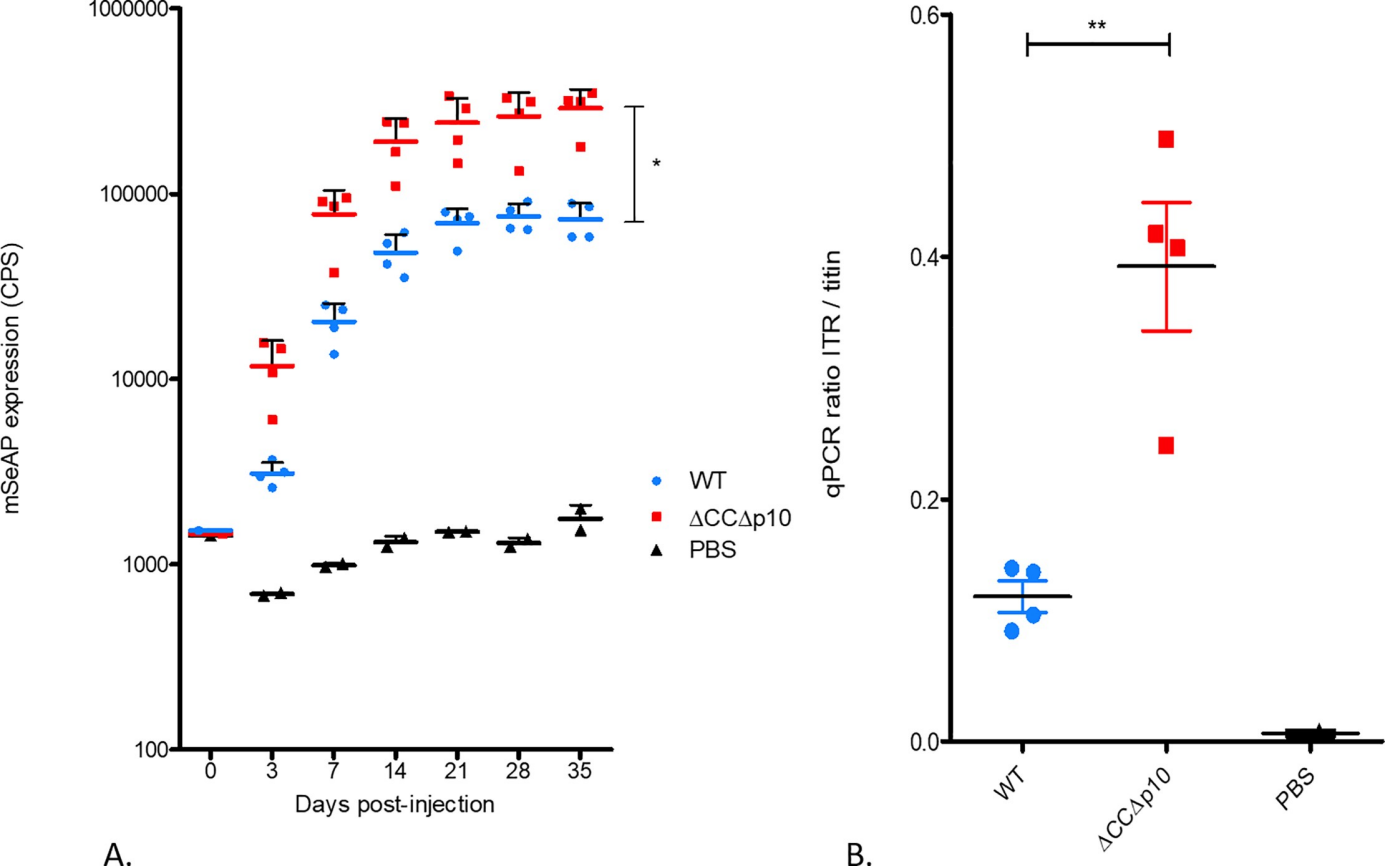
*In vivo* evaluation of rAAV vectors. rAAV8-mSeAP produced either using unmodified baculovirus (WT) or baculovirus with *v-cath* and *chiA* and *p10* genes inactivated (ΔCCΔp10). 10^9^ vg **of purified AAV8** were injected intramuscularly into mouse TA muscle (n = 4 per type of vector) and 2 mice were injected with PBS (as negative control). (A) Time course expression of seric mSeAP was measured on days 0, 2, 7, 14, 21, 28, and 35 post injection and is expressed as counts per second (* p<0.05). (B) The number of rAAV-mSeAP genome present in the injected muscles was measured and normalized to the level of the *titin* gene (cellular reference gene) (** p<0.01).

To easily monitor the impact of a lower level of full length VP1 in the rAAV8 capsid on the infectivity of the viral vector, we generated an rAAV8 encoding the mSeAP (murine Secreted Alkaline Phosphatase) reporter gene as transgene. rAAV8-mSeAP vectors were produced using the dual baculovirus system with or without deletions of the *chiA/v-cath* locus. In the latter construct we also had deleted the *p10* gene (AcbacΔCCΔp10). Both rAAV8 vectors were purified and injected into the Tibialis Anterior (TA) muscle of mice at a dose of 10^9^ vg per mouse. The mSeAP levels in serum samples were followed during 6 weeks after rAAV injections. We observed that the level of mSeAP measured in the murine sera reached a plateau 3 weeks post-injection (Fig 9A). The maximum mSeAP levels obtained were of 72835 counts per second (CPS) ± 16516 (std. dev.) of serum for the mice injected with the vector produced with the WT baculovirus 35 days post-injection and of 290853 CPS ± 76200 (std. dev.) with the ΔCCΔp10 baculovirus, hence an improvement by a factor 4 (t-test, p = 0.0158). This increase in the mSeAP level in murine sera correlated with a higher rAAV genome copy number present in the TA muscle myofibers (Fig 9B). The amount of rAAV-mSeAP gene copies compared to the cellular reference gene *titin* was 0.12 in mice treated with rAAV produced with the WT bacmid system and 0.39 when the rAAV was produced using the ΔCCΔp10 baculovirus (p<0.01).

## Discussion

We have studied the presence of supplementary capsid proteins in rAAV vectors produced with the baculovirus system and showed that their occurrence is caused to a large extent by the baculovirus protease v-CATH. While the deletion of the *v-cath* gene in conjunction with the *chiA* gene had been shown to enhance the productivity of recombinant proteins [11,24], the effect of their deletion on the production of complex biological products had not been studied. In the context of the production of rAAV vectors, the addition of the E64 protease inhibitor to the production cultures demonstrated that various rAAV serotypes were indeed susceptible to v-CATH activity. For susceptible serotypes, including rAAV1, 6, and 8 a major truncated VP protein of a size of 61 kDa was observed resulting from cleavage in the N-terminal VP1/2 unique domain after the Leu_189_-Gly_190_ motif located 13 amino acids upstream of the first amino acid of VP3. In rAAV8 a second potential v-CATH cleavage site was found in the VP1/2 unique domain following Leu_172_-Asn_173_. The difference in susceptibility between serotypes could be related to the presence or absence of specific amino acid sequences, that are present at the v-CATH cleavage site for rAAV1, 6, and 8, but absent in those rAAV serotypes that are non-susceptible to v-CATH cleavage (evaluated: rAAV2, 9, and rh10). These non-susceptible serotypes are characterized by replacement of Leu_189_ by Ile_189_ or Glu_191_ by Gln_191_. The substitution observed for rAAV serotypes 9 and rh10 appeared the be sufficient to abolish the cleavage, as the 10 amino acids located N- and C-terminally of this substitution were the same as for the susceptible AAV serotypes. When Leu_189_ was substituted by an Ile residue in the rAAV8 capsid, the cleavage at this position in the VP1/2 protein was indeed abolished. However, proteolytic activity of v-CATH was then observed at a second cleavage site, following amino acids Leu_172_-Asn_173_. The cleavage at this position might also have occurred in the non-mutated rAAV8, but may not have been visible on gel due to further trimming after Leu_189-_Gly_190_. However, the Glu_191_ to Gln_191_ mutation that we also performed did not abolish rAAV8 capsid degradation by v-CATH at this position, although such an amino acid replacement renders rAAV2 (almost) unsusceptible to v-CATH. It should be noted here, that in previous experiments, a small level of degradation could sometimes be observed at this position in the rAAV2 capsid.

Although the presence of Leu_189_ in VP1/2 seems to be an essential determinant for v-CATH susceptibility, rAAV8 seems to be the serotype most impacted by v-CATH activity when expressed with a WT baculovirus vector. In addition, the secondary cleavage site following Leu_172_-Asn_173_ amino acids was only found for rAAV8, despite the fact that the amino acid sequence of this VP region is highly conserved among the different AAV serotypes studied. Thus, amino acid sequence might not be the only parameter determining whether AAV is susceptible for degradation. Also secondary and tertiary structure variations in VP1/2 among the different serotypes might be important. Accessibility of the capsid proteins of the different serotypes to the protease should be considered along with the percentage of externalized VP1 and 2 proteins.

Interestingly, only full and not empty vector capsids of susceptible rAAV serotypes were cleaved by v-CATH activity. In addition, cleavage was independent of the size of the vector genome because cleavage could be observed for rAAV capsids harboring vector genomes of sizes, ranging from 845 nt (a small expression cassette used for the exon skipping strategy for treatment of Duchenne muscular dystrophy [25]) to 3867 nt (γSGC transgene), 4086 nt (recombinant mSeAP transgene), and 4679 nt (an undisclosed recombinant transgene, not shown). Previous work has shown that genome-containing AAV2 capsids underwent a conformational change upon heat shock, leading to the exposure of the N-terminal domains of VP1/VP2 [22]. This conformational change and exposure were probably triggered by the considerably increased internal pressure of full capsids, caused by the very high DNA packaging density [as reported for MVM parvoviruses [26]]. Thus, it appears logic that the same domains in rAAV8 are vulnerable to proteolytic degradation. The observation that the resulting small, 21 kDa N-terminal degradation product was not observed in any of the performed SDS-PAGE and Western blot analyses on rAAV particles, confirmed the idea that an exposed N-terminal end was attacked by v-CATH and subsequently lost from the rAAV particles.

However, the question remains at which moment and how this exposure of the N-terminal domain of VP1/VP2 and subsequent cleavage occurs. Based on previous observations, that on one side the inactive pro-v-cathepsin resides clustered with chitinase (as chaperon) in the Endoplasmic Reticulum [9], and that on the other side, rAAV capsid assembly takes place in vesicular bodies [27] in the nucleus (as observed for High Five cells), the cleavage of the N-terminus of VP1/VP2 cannot happen during synthesis of rAAV because of compartmental separation. Thus, cleavage can only occur after breakdown of the cellular compartments at the end of the baculoviral replication cycle and/or during/after release of the rAAV particles. In this context we could show that rAAV8 harvested from cultures with a high viability (>90%) did not show degradation of VP1/VP2 proteins (not shown). Degradation of the rAAV8 particles intensified at the end of the production process with the peak activity observed at the start of the purification process, initiated with the cell lysis. This last intensification of the degradation process could be linked to enhanced release and activation of the chitinase/cathepsin by detergent action and from efficient release of rAAV8 vectors from the cell culture bulk.

Transition from inside to outside of the capsids of the VP1 unique domain seems to be associated with endosomal pH and specific cellular factors [28]. Furthermore, reversible *in vitro* transition in the empty capsid structure at the 5-fold axis had been observed by lowering the pH from 7.5 to 6 [29] and could lead to the conformational change necessary for the VP1 externalization. At this pH transition, the VP1 unique domain underwent a reversible pH-induced unfolding/refolding process, accompanied by a loss/gain of the α-helical structure, that did not disrupt capsid integrity [30]. The reversible conformational changes in the VP1 unique domain likely facilitate the externalization of the N-terminal domain [30]. Based on these reports it can be hypothesized that there is a transient exposure of the N-terminal domains of VP1/VP2 molecules upon release of the rAAV particles into the cell culture medium that has a pH of about 6.0. It can be envisaged that a further reduction of the surrounding pH will shift the equilibrium to a higher fraction of exposed VP1/VP2 unique domains. There is however one aspect that is not entirely in favor of such a mechanism. Kronenberg et al. [22] presented a model in which the partially de-folded VP1-terminus was externalized upon heat shock related to amino acid residues 1–168. However, our experiments showed that the cleavage sites of v-CATH were situated at residue Leu_190_ (major cleavage site) and for rAAV8 also at position 173, signifying that according to Kronenberg et al. [22] these cleavage sites should be maintained inside the capsid. Further studies will be required to figure out the exact mechanism.

By comparing several rAAV serotypes we were able to demonstrate that another supplementary band (slightly smaller than the VP1 protein) was not a degradation product of the VP1 protein but originated from internal translation initiation in codon optimized *cap* sequences. Interestingly, we have previously shown that *rep* gene codon optimization could lead to supplementary transcription events compared to a non-optimized *rep* gene construct [31]. Thus codon optimization of AAV genes in the context of baculovirus expression system should be carefully checked and compared to non-optimized genes. This supplementary VP was incorporated into the AAV capsid and by reducing the level of the intact VP1 protein in the capsid, may potentially lower the infectivity of the rAAV vector. *In vivo* studies in mice using a model transgene (mSeAP) could confirm the importance of a complete and functional N-terminal region of VP1 for successful transduction. A fourfold increase in transduction efficiency was observed in comparison to rAAV8 vectors with a reduced VP1 integrity. The importance of the N-terminal domain of VP1 resides in the Phospho Lipase A2 (PLA2) domain that is involved in the endosomal escape of the (r)AAV during infection [28,32,33], allowing its nuclear trafficking. Obviously, degradation of the PLA2 domain should be precluded to maintain its functionality, either by using a delta-cathepsin baculovirus backbone or v-CATH inhibitors, such as E64. The latter option is useful under R&D conditions to understand the nature of the rAAV protein profiles, but application of E64 is not suitable for large scale production due to the associated costs and the necessity to prove its complete removal in the purification process. Thus, for rAAV serotypes susceptible to v-CATH, the use of a baculovirus with the *chiA*/*v-cath* locus deleted remains the best option for the production of the vectors. Baculovirus systems with only *chiA* deletions might also be successful, as these produce only inactive pro-v-CATH [34], but that was not tested in this study.

In conclusion, we could show that the removal or inactivation of baculoviral v-CATH protease led to improved VP1 integrity for rAAV serotypes 1, 6, and 8 and a higher level of transduction by rAAV8. Moreover, using non-codon optimized nucleotide sequences (except for the start codon) will further improve the infectivity of rAAV serotypes 1, 6, and 8. In this way the generation of truncated VP1 proteins that serve as potential competitors for functional VP1 is prevented. The implementation of these improvements will lead to a reduction of the overall vector dose to be administered to the patients, which is of medical and economic importance.

## Supporting information

S1 Fig Cap ORFs used in this study. VP1 ATG start codon is replaced by ACG in all *cap* ORFs. *Cap1* and *cap6* ORF are corresponding to the wild type sequences. Both *cap8* WT and codon optimized ORF sequences are presented. *Cap2*, *cap9* and *cap rh10* are codon-optimized versions.
(PDF)

S1 Table Primers and probes used in this study. (PDF)
