# Supplementary material for: Origins of truncated supplementary capsid proteins in rAAV8 vectors produced with the baculovirus system
Source: PLoS One. 2018 Nov 15;13(11):e0207414. doi: 10.1371/journal.pone.0207414 (PMC6237368; doi:10.1371/journal.pone.0207414)
Supplement: S1 Fig — VP1 ATG start codon is replaced by ACG in all cap ORFs. Cap1 and cap6 ORF are corresponding to the wild type sequences. Both cap8 WT and codon optimized ORF sequences are presented. Cap2, cap9 and cap rh10 are codon-optimized versions. (PDF) [file pone.0207414.s001.pdf]

S1 Fig.

> cap1 (similar to WT sequence)

acggctgccgatggttatcttccagattggctcgaggacaacctctctgagggcattcgcgagtggtgggacttgaaacctggagccccgaagcccaaagc
caaccagcaaaagcaggacgacggccggggtctggtgcttcctggctacaagtacctcggacccttcaacggactcgacaagggggagcccgtcaacgc
ggcggacgcagcggccctcgagcacgacaaggcctacgaccagcagctcaaagcgggtgacaatccgtacctgcggtataaccacgccgacgccgagtt
tcaggagcgtctgcaagaagatacgtcttttgggggcaacctcgggcgagcagtcttccaggccaagaagcgggttctcgaacctctcggtctggttgagg
aaggcgctaagacggctcctggaaagaaacgtccggtagagcagtcgccacaagagccagactcctcctcgggcatcggcaagacaggccagcagccc
gctaaaaagagactcaattttggtcagactggcgactcagagtcagtccccgatccacaacctctcggagaacctccagcaaccccgctgctgtgggacc
tactacaatggcttcaggcggtggcgcaccaatggcagacaataacgaaggcgccgacggagtgggtaatgcctcaggaaattggcattgcgattccaca
tggctgggcgacagagtcatcaccaccagcacccgcacctgggccttgcccacctacaataaccacctctacaagcaaatctccagtgcttcaacgggggc
cagcaacgacaaccactacttcggctacagcacccccctgggggtattttgatttcaacagattccactgccacttttcaccacgtgactggcagcgactcatc
aacaacaattggggattccggcccaagagactcaacttcaaactcttcaacatccaagtcaaggaggtcacgacgaatgatggcgtcacaaccatcgcta
ataaccttaccagcacggttcaagtcttctcggactcggagtaccagcttccgtacgtcctcggctctgcgcaccagggctgcctccctccgttcccggcgga
cgtgttcatgattccgcaatacggctacctgacgctcaacaatggcagccaagccgtgggacgttcatccttttactgcctggaatatttcccttctcagatgc
tgagaacgggcaacaactttaccttcagctacacctttgaggaagtgcctttccacagcagctacgcgcacagccagagcctggaccggctgatgaatcct
ctcatcgaccaatacctgtattacctgaacagaactcaaaatcagtccggaagtgcccaaaacaaggacttgctgtttagccgtgggtctccagctggcatg
tctgttcagcccaaaaactggctacctggaccctgttatcggcagcagcgcgtttctaaaacaaaaacagacaacaacaacagcaattttacctggactgg
tgcttcaaaatataacctcaatgggcgtgaatccatcatcaaccctggcactgctatggcctcacacaaagacgacgaagacaagttctttcccatgagcg
gtgtcatgatttttggaaaagagagcgccggagcttcaaacactgcattggacaatgtcatgattacagacgaagaggaaattaaagccactaaccctgtg
gccaccgaaagatttgggaccgtggcagtcaatttccagagcagcagcacagaccctgcgaccggagatgtgcatgctatgggagcattacctggcatgg
tgtggcaagatagagacgtgtacctgcagggtcccatttgggccaaaattcctcacacagatggacactttcacccgtctcctcttatgggcggctttggact
caagaacccgcctcctcagatcctcatcaaaaacacgcctgttcctgcgaatcctccggcggagttttcagctacaaagtttgcttcattcatcacccaatac
tccacaggacaagtgagtgtggaaattgaatgggagctgcagaaagaaaacagcaagcgctggaatcccgaagtgcagtacacatccaattatgcaaa
atctgccaacgttgattttactgtggacaacaatggactttatactgagcctcgccccattggcacccgttaccttacccgtcccctgtaa

> cap2 (codon-optimized)

acggctgccgacggttatctacccgattggctcgaggacactctctctgaaggaataagacagtggtggaagctcaaacccggcccaccaccaccaaagc
ccgcagagcggcataaggacgacagcaggggccttgtgcttcccgggtacaagtacctcggacccttcaacggactggacaagggagagccggtgaacg
aggcagacgccgctgccctcgagcacgacaaagcctacgaccggcagctcgacagcggagacaacccttacctcaagtacaaccacgccgacgcggag
ttccaggagcgccttaaagaagatacgtcttttgggggcaacctcggacgagcagtcttccaggcgaaaaagagggttcttgaacctctgggcctggttga
ggaacctgttaagacggctccgggaaaaaagaggccggtagagcactctcctgtggagccagactcctcctcgggaaccggaaaggcgggccagcagcc
tgcaagaaaaagattgaattttggtcagactggagacgcagactcagtacctgacccccagcctctcggacagccaccagcagcccccctctggtctggga
actaatacgatggctacaggcagtggcgcaccaatggcagacaataacgagggcgccgacggagtgggtaattcctcgggaaattggcattgcgattcca
catggatgggcgacagagtcatcaccaccagcacccgaacctgggccctgcccacctacaacaaccacctctacaaacaaatttccagccaatcaggagc
ctcgaacgacaatcactactttggctacagcaccccttgggggtattttgacttcaacagattccactgccacttttcaccacgtgactggcaaagactcatc
aacaacaactggggattccgacccaagagactcaacttcaagctctttaacattcaagtcaaagaggtcacgcagaatgacggtacgacgacgattgcca
ataaccttaccagcacggttcaggtgtttactgactcggagtaccagctcccgtacgtcctcggctcggcgcatcaaggatgcctcccgccgttcccagcag
acgtcttcatggtgccacagtatggatacctcaccctgaacaacgggagtcaggcagtaggacgctcttcattttactgcctggagtactttccttctcagat
gctgcgtaccggaaacaactttaccttcagctacacttttgaggacgttcctttccacagcagctacgctcacagccagagtctggaccgtctcatgaatcct
ctcatcgaccagtacctgtattacttgagcagaacaaacactccaagtggaaccaccacgcagtcaaggcttcagttttctcaggccggagcgagtgacat
tcgggaccagtctaggaactggcttcctggaccctgttaccgccagcagcgagtatcaaagacatctgcggataacaacaacagtgaatactcgtggactg
gagctaccaagtaccacctcaatggcagagactctctggtgaatccgggcccggccatggcaagccacaaggacgatgaagaaaagtttttcctcagag

cggggttctcatctttgggaagcaaggctcagagaaaacaaatgtggacattgaaaaggtcatgattacagacgaagaggaaatcaggacaaccaatcc
cgtggctacggagcagtatggttctgtatctaccaacctccagagaggcaacagacaagcagctaccgcagatgtcaacacacaaggcgttcttccaggc
atggtctggcaggacagagatgtgtaccttcaggggcccatctgggcaaagattccacacacggacggacattttcacccctctcccctcatgggtggattc
ggacttaaacaccctcctccacagattctcatcaagaacaccccggtacctgcgaatccttcgaccaccttcagtgcggcaaagtttgcttccttcatcacac
agtactccacgggacaggtcagcgtggagatcgagtgggagctgcagaaggaaaacagcaaacgctggaatcccgaaattcagtacacttccaactaca
acaagtctgttaatgtggactttactgtggacactaatggcgtgtattcagagcctcgccccattggcaccagatacctgactcgtaatctgtaa

> cap6 (similar to WT sequence)

acggctgccgatggttatcttccagattggctcgaggacaacctctctgagggcattcgcgagtggtgggacttgaaacctggagccccgaaacccaaagc
caaccagcaaaagcaggacgacggccggggtctggtgcttcctggctacaagtacctcggacccttcaacggactcgacaagggggagcccgtcaacgc
ggcggatgcagcggccctcgagcacgacaaggcctacgaccagcagctcaaagcgggtgacaatccgtacctgcggtataaccacgccgacgccgagtt
tcaggagcgtctgcaagaagatacgtcttttgggggcaacctcgggcgagcagtcttccaggccaagaagagggttctcgaaccttttggtctggttgagg
aaggtgctaagacggctcctggaaagaaacgtccggtagagcagtcgccacaagagccagactcctcctcgggcattggcaagacaggccagcagcccg
ctaaaaagagactcaattttggtcagactggcgactcagagtcagtccccgacccacaacctctcggagaacctccagcaaccccgctgctgtgggacct
actacaatggcttcaggcggtggcgcaccaatggcagacaataacgaaggcgccgacggagtgggtaatgcctcaggaaattggcattgcgattccacat
ggctgggcgacagagtcatcaccaccagcacccgaacatgggccttgcccacctataacaaccacctctacaagcaaatctccagtgcttcaacgggggc
cagcaacgacaaccactacttcggctacagcacccccctgggggtattttgatttcaacagattccactgccatttctcaccacgtgactggcagcgactcatc
aacaacaattggggattccggcccaagagactcaacttcaagctcttcaacatccaagtcaaggaggtcacgacgaatgatggcgtcacgaccatcgcta
ataaccttaccagcacggttcaagtcttctcggactcggagtaccagttgccgtacgtcctcggctctgcgcaccagggctgcctccctccgttcccggcgga
cgtgttcatgattccgcagtacggctacctaacgctcaacaatggcagccaggcagtgggacggtcatccttttactgcctggaatatttcccatcgcagatg
ctgagaacgggcaataactttaccttcagctacaccttcgaggacgtgcctttccacagcagctacgcgcacagccagagcctggaccggctgatgaatcc
tctcatcgaccagtacctgtattacctgaacagaactcagaatcagtccggaagtgcccaaaacaaggacttgctgtttagccgggggtctccagctggcat
gtctgttcagcccaaaaactggctacctggaccctgttaccggcagcagcgcgtttctaaaacaaaaacagacaacaacaacagcaactttacctggactg
gtgcttcaaaatataaccttaatgggcgtgaatctataatcaaccctggcactgctatggcctcacacaaagacgacaaagacaagttctttcccatgagcg
gtgtcatgatttttggaaaggagagcgccggagcttcaaacactgcattggacaatgtcatgatcacagacgaagaggaaatcaaagccactaaccccgt
ggccaccgaaagatttgggactgtggcagtcaatctccagagcagcagcacagaccctgcgaccggagatgtgcatgttatgggagccttacctggaatg
gtgtggcaagacagagacgtatacctgcagggtcctatttggccaaaattcctcacacggatggacactttcacccgtctcctctcatgggcggctttgga
cttaagcacccgcctcctcagatcctcatcaaaaacacgcctgttcctgcgaatcctccggcagagttttcggctacaaagtttgcttcattcatcacccagta
ttccacaggacaagtgagcgtggagattgaatgggagctgcagaaagaaaacagcaaacgctggaatcccgaagtgcagtatacatctaactatgcaaa
atctgccaacgttgatttcactgtggacaacaatggactttatactgagcctcgccccattggcacccgttacctcacccgtcccctgtaa

> cap8 (codon-optimized)

acggctgccgacggttatctacccgattggctcgaggacaacctctctgagggcattcgcgagtggtgggccctcaaacccggcgctcctaagccaaaggc
taaccaacagaaacaagacgacggacgcggcctggttctccccggttacaaatacctaggacccttcaacgggctggacaagggcgaacccgtgaacgc
tgcggacgccgctgcccttgagcacgataaggcttacgaccagcaactgcaggccggcgacaacccttaccttcgttataaccacgccgacgctgaattcc
aggagcgtctgcaagaagatacgtcttttgggggcaacctcgggcgagcagtcttccaggccaagaagcgggttctcgaacctctcggtctggttgaggaa
ggcgctaagacggctcctggaaagaagagaccggtagagccatcaccccagcgttctccagactcctctacgggcatcggcaagaaaggccaacagccc
gccagaaaaagactcaattttggtcagactggcgactcagagtcagttccagaccctcaacctctcggagaacctccagcagcgccctctggtgtgggacc
taatacaatggctgcaggcggtggcgcaccaatggcagacaataacgaaggcgccgacggagtgggtagttcctcgggaaattggcattgcgattccaca
tggctgggcgacagagtcatcaccaccagcacccgaacctgggccctgcccacctacaacaaccacctctacaagcaaatctccaacgggacatcggga
ggagccaccaacgacaacacctacttcggctacagcacccccctgggggtattttgactttaacagattccactgccacttttcaccacgtgactggcagcga
ctcatcaacaacaactggggattccggcccaagagactcagcttcaagctcttcaacatccaggtcaaggaggtcacgcagaatgaaggcaccaagacc
atcgccaataacctcaccagcaccatccaggtgtttacggactcggagtaccagctgccgtacgttctcggctctgcccaccagggctgcctgcctccgttcc
cggcggacgtgttcatgattccccagtacggctacctaacactcaacaacggtagtcaggccgtgggacgctcctccttctactgcctggaatactttccttc
gcagatgctgagaaccggcaacaacttccagtttacttacaccttcgaggacgtgcctttccacagcagctacgcccacagccagagcttggaccggctga

tgaatcctctgattgaccagtacctgtactacttgtctcggactcaaacaacaggaggcacggcaaatacgcagactctgggcttcagccaaggtgggcct
aatacaatggccaatcaggcaaagaactggctgccaggaccctgttaccgccaacaacgcgtctcaacgacaaccgggcaaaacaacaatagcaacttt
gcctggactgctgggaccaaataccatctgaatggaagaaattcattggctaatcctggcatcgctatggcaacacacaaagacgacgaggagcgtttttt
tcccagtaacgggatcctgatttttggcaaacaaaatgctgccagagacaatgcggattacagcgatgtcatgctcaccagcgaggaagaaatcaaaacc
actaaccctgtggctacagaggaatacggtatcgtggcagataacttgcagcagcaaaacacggctcctcaaattggaactgtcaacagccagggggcct
tacccggtatggtctggcagaaccgggacgtgtacctgcagggtcccatctgggccaagattcctcacacggacggcaacttccacccgtctccgctgatg
ggcggctttggcctgaaacatcctccgcctcagatcctgatcaagaacacgcctgtacctgcggatcctccgaccaccttcaaccagtcaaagctgaactct
ttcatcacgcaatacagcaccggacaggtcagcgtggaaattgaatgggagctgcagaaggaaaacagcaagcgctggaaccccgagatccagtacac
ctccaactactacaaatctacaagtgtggactttgctgttaatacagaaggcgtgtactctgaaccccgccccattggcacccgttacctcacccgtaatctg
taa

> cap8 (similar to WT sequence)

acggctgccgatggttatcttccagattggctcgaggacaacctctctgagggcattcgcgagtggtgggcgctgaaacctggagccccgaagcccaaagc
caaccagcaaaagcaggacgacggccggggtctggtgcttcctggctacaagtacctcggacccttcaacggactcgacaagggggagcccgtcaacgc
ggcggacgcagcggccctcgagcacgacaaggcctacgaccagcagctgcaggcgggtgacaatccgtacctgcggtataaccacgccgacgccgagtt
tcaggagcgtctgcaagaagatacgtcttttgggggcaacctcgggcgagcagtcttccaggccaagaagcgggttctcgaacctctcggtctggttgagg
aaggcgctaagacggctcctggaaagaagagaccggtagagccatcaccccagcgttctccagactcctctacgggcatcggcaagaaaggccaacagc
ccgccagaaaaagactcaattttggtcagactggcgactcagagtcagttccagaccctcaacctctcggagaacctccagcagcgccctctggtgtggga
cctaatacaatggctgcaggcggtggcgcaccaatggcagacaataacgaaggcgccgacggagtgggtagttcctcgggaaattggcattgcgattcca
catggctgggcgacagagtcatcaccaccagcacccgaacctgggccctgcccacctacaacaaccacctctacaagcaaatctccaacgggacatcggg
aggagccaccaacgacaacacctacttcggctacagcaccccctgggggtattttgactttaacagattccactgccacttttcaccacgtgactggcagcg
actcatcaacaacaactggggattccggcccaagagactcagcttcaagctcttcaacatccaggtcaaggaggtcacgcagaatgaaggcaccaagac
catcgccaataacctcaccagcaccatccaggtgtttacggactcggagtaccagctgccgtacgttctcggctctgcccaccagggctgcctgcctccgttc
ccggcggacgtgttcatgattccccagtacggctacctaacactcaacaacggtagtcaggccgtgggacgctcctccttctactgcctggaatactttcctt
cgcagatgctgagaaccggcaacaacttccagtttacttacaccttcgaggacgtgcctttccacagcagctacgcccacagccagagcttggaccggctg
atgaatcctctgattgaccagtacctgtactacttgtctcggactcaaacaacaggaggcacggcaaatacgcagactctgggcttcagccaaggtgggcc
taatacaatggccaatcaggcaaagaactggctgccaggaccctgttaccgccaacaacgcgtctcaacgacaaccgggcaaaacaacaatagcaactt
tgcctggactgctgggaccaaataccatctgaatggaagaaattcattggctaatcctggcatcgctatggcaacacacaaagacgacgaggagcgttttt
ttcccagtaacgggatcctgatttttggcaaacaaaatgctgccagagacaatgcggattacagcgatgtcatgctcaccagcgaggaagaaatcaaaac
cactaaccctgtggctacagaggaatacggtatcgtggcagataacttgcagcagcaaaacacggctcctcaaattggaactgtcaacagccagggggcc
ttacccggtatggtctggcagaaccgggacgtgtacctgcagggtcccatctgggccaagattcctcacacggacggcaacttccacccgtctccgctgatg
ggcggctttggcctgaaacatcctccgcctcagatcctgatcaagaacacgcctgtacctgcggatcctccgaccaccttcaaccagtcaaagctgaactct
ttcatcacgcaatacagcaccggacaggtcagcgtggaaattgaatgggagctgcagaaggaaaacagcaagcgctggaaccccgagatccagtacac
ctccaactactacaaatctacaagtgtggactttgctgttaatacagaaggcgtgtactctgaaccccgccccattggcacccgttacctcacccgtaatctg
taa

> cap9 (codon-optimized)

acggctgccgacggttatctacccgattggctcgaggacaacctttctgaaggaattcgcgagtggtgggctctcaaacctggagcccctcaacccaaggc
aaatcaacaacatcaagacaacgctcgaggtcttgtgcttccgggttacaaataccttggacccggcaacggactcgacaagggggagccggtcaacgca
gcagacgcggcggccctcgagcacgacaaggcctacgaccagcagctcaaggccggagacaacccgtacctcaagtacaaccacgccgacgccgagtt
ccaggagcggctcaaagaagatacgtcttttgggggcaacctcgggcgagcagtcttccaggccaaaaagaggcttcttgaacctcttggtctggttgagg
aagcggctaagacggctcctggaaagaagaggcctgtagagcagtctcctcaggaaccggactcctccgcgggtattggcaaatcgggtgcacagcccg
ctaaaaagagactcaatttcggtcagactggcgacacagagtcagtcccagaccctcaaccaatcggagaacctcccgcagccccctcaggtgtgggatc
tcttacaatggcttcaggtggtggcgcaccagtggcagacaataacgaaggtgccgatggagtgggtagttcctcgggaaattggcattgcgattcccaat
ggctgggggacagagtcatcaccaccagcacccgaacctgggccctgcccacctacaacaatcacctctacaagcaaatctccaacagcacatctggagg
atcttcaaatgacaacgcctacttcggctacagcaccccctgggggtattttgacttcaacagattccactgccacttctcaccacgtgactggcagcgactc

atcaacaacaactggggattccggcctaagcgactcaacttcaagctcttcaacattcaggtcaaagaggttacggacaacaatggagtcaagaccatcg
ccaataaccttaccagcacggtccaggtcttcacggactcagactatcagctcccgtacgtgctcgggtcggctcacgagggctgcctcccgccgttcccag
cggacgttttcatgattcctcagtacgggtatctgacgcttaatgatggaagccaggccgtgggtcgttcgtccttttactgcctggaatatttcccgtcgcaa
atgctaagaacgggtaacaacttccagttcagctacgagtttgagaacgtacctttccatagcagctacgctcacagccaaagcctggaccgactaatgaa
tccactcatcgaccaatacttgtactatctctcaaagactattaacggttctggacagaatcaacaaacgctaaaattcagtgtggccggacccagcaacat
ggctgtccagggaagaaactacatacctggacccagctaccgacaacaacgtgtctcaaccactgtgactcaaaacaacaacagcgaatttgcttggcct
ggagcttcttcttgggctctcaatggacgtaatagcttgatgaatcctggacctgctatggccagccacaaagaaggagaggaccgtttctttcctttgtctg
gatctttaatttttggcaaacaaggaactggaagagacaacgtggatgcggacaaagtcatgataaccaacgaagaagaaattaaaactactaacccgg
tagcaacggagtcctatggacaagtggccacaaaccaccagagtgcccaagcacaggcgcagaccggctgggttcaaaaccaaggaatacttccgggta
tggtttggcaggacagagatgtgtacctgcaaggacccatttgggccaaaattcctcacacggacggcaactttcacccttctccgctgatgggagggtttg
gaatgaagcacccgcctcctcagatcctcatcaaaaacacacctgtacctgcggatcctccaacggccttcaacaaggacaagctgaactctttcatcacc
cagtattctactggccaagtcagcgtggagatcgagtgggagctgcagaaggaaaacagcaagcgctggaacccggagatccagtacacttccaactatt
acaagtctaataatgttgaatttgctgttaatactgaaggtgtatatagtgaaccccgccccattggcaccagatacctgactcgtaatctgtaa

>cap rh10 (codon-optimized)

acggctgccgacggttatctacccgattggctcgaggacaacctctctgagggcattcgcgagtggtgggacctgaaacccggcgctccgaaacccaaag
ccaaccaacagaaacaagacgacggccgcggtctggtgctgcccggctacaaatacctgggacccttcaacggcctggacaagggtgaacccgtgaacg
ccgcggacgccgcggccttggagcacgacaaggcctacgaccagcaactcaaagcgggcgacaacccgtacctgcgctataaccacgccgacgcggaa
tttcaggagcgtctgcaagaagatacgtcttttgggggcaacctcgggcgagcagtcttccaggccaagaagcgggttctcgaacctctcggtctggttgag
gaaggcgctaagacggctcctggaaagaagagaccggtagagccatcaccccagcgttctccagactcctctacgggcatcggcaagaaaggccagcag
cccgcgaaaaagagactcaactttgggcagactggcgactcagagtcagtgcccgaccctcaaccaatcggagaacccccgcaggcccctctggtctgg
gatctggtacaatggctgcaggcggtggcgctccaatggcagacaataacgaaggcgccgacggagtgggtagttcctcaggaaattggcattgcgattc
cacatggctgggcgacagagtcatcaccaccagcacccgaacctgggccctccccacctacaacaaccacctctacaagcaaatctccaacgggacttcg
ggaggaagcaccaacgacaacacctacttcggctacagcaccccctgggggtattttgactttaacagattccactgccacttctcaccacgtgactggcag
cgactcatcaacaacaactggggattccggcccaagagactcaacttcaagctcttcaacatccaggtcaaggaggtcacgcagaatgaaggcaccaag
accatcgccaataaccttaccagcacgattcaggtctttacggactcggaataccagctcccgtacgtcctcggctctgcgcaccagggctgcctgcctccgt
tcccggcggacgtcttcatgattcctcagtacgggtacctgactctgaacaatggcagtcaggccgtgggccgttcctccttctactgcctggagtactttcct
tctcaaatgctgagaacgggcaacaactttgagttcagctaccagtttgaggacgtgccttttcacagcagctacgcgcacagccaaagcctggaccggct
gatgaacccctcatcgaccagtacctgtactacctgtctcggactcagtccacgggaggtaccgcaggaactcagcagttgctattttctcaggccgggcc
taataacatgtcggctcaggccaaaaactggctacccgggccctgctaccggcagcaacgcgtctccacgacactgtcgcaaaataacaacagcaacttt
gcctggaccggtgccaccaagtatcatctgaatggcagagactctctggtaaatcccggtgtcgctatggcaacccacaaggacgacgaagagcgatttt
tccgtccagcggagtcttaatgtttgggaaacagggagctggaaaagacaacgtggactatagcagcgttatgctaaccagtgaggaagaaattaaaacc
accaacccagtggccacagaacagtacggcgtggtggccgataacctgcaacagcaaaacgccgctcctattgtaggggccgtcaacagtcaaggagcc
ttacctggcatggtctggcagaaccgggacgtgtacctgcagggtcctatctgggccaagattcctcacacggacggaaactttcatccctcgccgctgatg
ggaggctttggactgaaacacccgcctcctcagatcctgattaagaatacacctgttcccgcggatcctccaactaccttcagtcaagctaagctggcgtcg
ttcatcacgcagtacagcaccggacaggtcagcgtggaaattgaatgggagctgcagaaagaaaacagcaaacgctggaacccagagattcaatacact
tccaactactacaaatctacaaatgtggactttgctgttaacacagatggcacttattctgagcctcgccccatcggcacccgttacctcacccgtaatctgta
a
