# Supplementary material for: Origins of truncated supplementary capsid proteins in rAAV8 vectors produced with the baculovirus system
Source: PLoS One. 2018 Nov 15;13(11):e0207414. doi: 10.1371/journal.pone.0207414 (PMC6237368; doi:10.1371/journal.pone.0207414)
Supplement: S1 Table — (PDF) [file pone.0207414.s002.pdf]

Primer	Sequence 5’ to 3’	Purpose*
CC-KO-F	CCGCTGTTGAAACAATATTTTATAATACCCTGTTTATAGTTAACAATGTCGGCAGCGTCTATGGCCATAGGAATAGGGCCTACCGTTCGTATAATGTATGCTATACGAAGTTAT	chitinase/cathepsin gene deletion nt 105771- 107700
CC-KO-R	CCGCTGTTGAAACAATATTTTATAATACCCTGTTTATAGTTAACAATGTCGGCAGCGTCTATGGCCATAGGAATAGGGCCTACCGTTCGTATAATGTATGCTATACGAAGTTAT	chitinase/cathepsin gene deletion nt 105771- 107700
chitinase-105625F	CGCGGCCGTACATGGCGACGCCCA	Gene KO Verification
cathepsin-107849R	GTTTTTAAAGGTCCAATATGGAATG	Gene KO Verification
p10-KO-F	TTGTATATTAATTAAAATACTATACTGTAAATTACATTTTATTTACAATCTACCGTTCGTATAGCATACATTATACGAAGTTAT	p10 ORF KO nt 118839 - 119121
P10-KO-R	GAATCGTACGAATATTATAAAACAATTGATTTGTTATTTTAAAAACGATTTACCGTTCGTATAATGTATGCTATACGAAGTTAT	p10 ORF KO nt 118839 - 119121
p10-118725-F	CCGGGACCTTTAATTCAACCCAACA	Gene KO Verification
p10-119259-R	CAGCATTTGTTATACACACAGAACT	Gene KO Verification
M13pucF	CCAGTCACGACGTTGTAAAACG	Verification of transposed bacmid
M13pucR	AGCGGATAACAATTTCACACAGG	Verification of transposed bacmid
Genta	AGCCACCTACTCCCAACATC	Verification of transposed bacmid
ITR-F	CTCCACTAGGGGTTCCTTG	QPCR in rAAV ITR
ITR-R	GTAGATAAGTAGCATGGC	QPCR in rAAV ITR
ITR-P	[FAM] TAGTTAATGATTAACCC [MGB-NFQ]	QPCR probe in rAAV ITR
titin-F	AAAACGAGCAGTGACGTGAGC	Mus musculus titin gene QPCR
titin-R	TTCAGTCATGCTGCTAGCGC	Mus musculus titin gene QPCR
titin-P	[VIC] TGCACGGAAGCGTCTCGTCTCAGTC [TAMRA]	Mus musculus titin gene QPCR probe
VP1-KO-F	ggattcctgccgccacTgctgccgacggttatctacccgattggc	AAV2 VP1 silencing**
VP1-KO-R	ccaatcgggtagataaccgtcggcagcAgtggcggcaggaatcc	AAV2 VP1 silencing**
VP2-KO-F	cctggttgaggaacctgttaagacCgctccgggaaaaaagaggccggtagagcactctcc	AAV2 VP2 silencing**
VP2-KO-R	ggagagtgctctaccggcctcttttttcccggagcGgtcttaacaggttcctcaaccagg	AAV2 VP2 silencing**

* Baculovirus numbering is performed accordingly to Ayres et al., 1994. FAM: 6-carboxyfluorescein. TAMRA: tetramethylrhodamine. MGB-NFQ: Minor Grove Binding-Non Fluorescent Quencher
** Upper case correspond to modified nucleic acids
